# Role of Artificial Intelligence and Machine Learning in Diagnosing Knee Lesions: Where Are We Now?

**DOI:** 10.7759/cureus.106869

**Published:** 2026-04-12

**Authors:** Paraskevas-Asimakis Velitsikakis, Pavlos Altsitzioglou, Dimitrios Serenidis, Orestis Konstantas, Panagiotis Koulouvaris, Dimitrios S Mastrokalos, Dimitrios Koulalis

**Affiliations:** 1 1st Department of Orthopaedic Surgery, National and Kapodistrian University Athens, “Attikon” Hospital, Athens, GRC

**Keywords:** artificial intelligence, knee lesions, machine learning, medical imaging, mri

## Abstract

Globally, knee lesions, including anterior cruciate ligament (ACL), menisci, cartilage, and osteoarthritis, are a major clinical burden. Clinical examination and medical imaging are key diagnostic methods, but they are inaccurate, inefficient, and rarely reproducible. Musculoskeletal imaging diagnostic precision and workflows may improve with artificial intelligence (AI) and machine learning (ML) technologies. To comprehensively review AI and ML applications for knee lesion diagnosis, compare their diagnostic performance to conventional methods, and identify key challenges and future clinical directions, a systematic literature review was conducted on PubMed, Scopus, and IEEE Xplore for studies from 2015 to the present. Keywords included "artificial intelligence", "machine learning", "deep learning", "knee", "MRI", "diagnosis", and "lesion detection". Performance metrics, methodological rigor, and relevance to AI/ML knee lesion diagnosis were considered when selecting studies. Analyses included peer-reviewed articles and regulatory guidance. There were 14 AI/ML knee lesion diagnosis studies in the review. Convolutional neural networks (CNNs) were the most popular architecture, with ResNet, VGG, DenseNet, and custom architectures performing well. Deep learning models were accurate diagnostically. AI-assisted diagnosis excelled at ACL tear, osteoarthritis grading, and meniscal tear detection. AI systems outperformed radiologists in diagnostic accuracy, interpretation time, and inter-reader agreement. In many applications, AI and ML technologies can diagnose knee lesions with human-level accuracy. However, dataset diversity, model generalizability, regulatory approval, and clinical integration remain issues. Clinical deployment requires ongoing research on standardized protocols, diverse training datasets, and real-world validation.

## Introduction and background

Knee lesions encompass a broad spectrum of pathological conditions affecting one of the body's most complex joints, including traumatic injuries, degenerative diseases, and inflammatory conditions. The epidemiological burden of knee pathology is substantial, with knee osteoarthritis alone affecting approximately 374.74 million people globally as of 2021, representing an age-standardized prevalence rate of 4,294.27 per 100,000 population [1]. Sports-related knee injuries, particularly anterior cruciate ligament (ACL) tears and meniscal injuries, occur at rates of 4.06 per 1000 game exposures in professional athletics, with significant implications for both short-term performance and long-term joint health [2].

The current diagnostic approach for knee lesions relies primarily on clinical examination combined with imaging, including radiography, magnetic resonance imaging (MRI), computed tomography (CT), and ultrasound [3]. While MRI has emerged as the gold standard for soft tissue evaluation of the knee, achieving sensitivity and specificity rates of 87% and 93% for ACL tears, 89% and 88% for medial meniscal tears, and 78% and 95% for lateral meniscal tears, respectively, several limitations persist [4]. These include inter-observer variability, time-intensive interpretation processes, and suboptimal sensitivity for early-stage pathological changes [5].

The integration of artificial intelligence (AI) and machine learning (ML) into medical imaging has emerged as a transformative approach to address these limitations [6]. AI technologies, particularly deep learning algorithms, have demonstrated remarkable capabilities in pattern recognition, image segmentation, and diagnostic classification tasks. In the context of knee imaging, these technologies offer the potential to enhance diagnostic accuracy, reduce interpretation time, standardize reporting practices, and identify subtle pathological changes that may be overlooked by traditional visual assessment [7].

Recent advances in computational power, the availability of large-scale medical imaging datasets, and sophisticated algorithm development have brought significant progress in AI-driven diagnostic tools for musculoskeletal imaging [8]. The U.S. Food and Drug Administration (FDA) has authorized nearly 1,000 AI-enabled medical devices as of 2023, with more than 75% dedicated to radiology applications, underscoring the clinical relevance and regulatory acceptance of these technologies [9].

This comprehensive review aims to provide a current assessment of AI and ML applications in knee lesion diagnosis, bringing together evidence from recent studies to evaluate diagnostic performance, identify key technological approaches, and discuss the challenges and opportunities that lie ahead for clinical implementation. By examining the state-of-the-art in this rapidly evolving field, we seek to inform clinicians, researchers, and healthcare administrators about the current capabilities and future potential of AI-driven diagnostic tools in knee pathology.

## Review

Methods

Search Strategy

A comprehensive systematic literature search was conducted across multiple electronic databases, including PubMed/MEDLINE, Scopus, IEEE Xplore, and Google Scholar. The search strategy employed both Medical Subject Headings (MeSH) terms and free-text keywords to maximize the retrieval of relevant literature. Primary search terms included combinations of "artificial intelligence", "machine learning", "deep learning", "convolutional neural network", "knee", "MRI", "magnetic resonance imaging", "diagnosis", "lesion detection", "anterior cruciate ligament", "ACL", "meniscus", "cartilage", "osteoarthritis", and "diagnostic accuracy".

The Boolean search strategy utilized the following structure: (("artificial intelligence" OR "machine learning" OR "deep learning" OR "neural network") AND ("knee" OR "ACL" OR "meniscus" OR "cartilage") AND ("MRI" OR "magnetic resonance" OR "imaging") AND ("diagnosis" OR "detection" OR "classification")).

The database search yielded a total of 512 records across PubMed, Scopus, IEEE Xplore, and Google Scholar. After removal of 275 records for the following reasons: lack of quantitative diagnostic performance metrics, conference abstracts without full-text, non-clinical or technical focus without diagnostic application, review articles without original data, or insufficient methodological detail, a total of 237 full-text articles were assessed for eligibility. Of these, 223 records were excluded based on being clinically irrelevant, non-English, or too old (before 2015).

**Figure 1:**
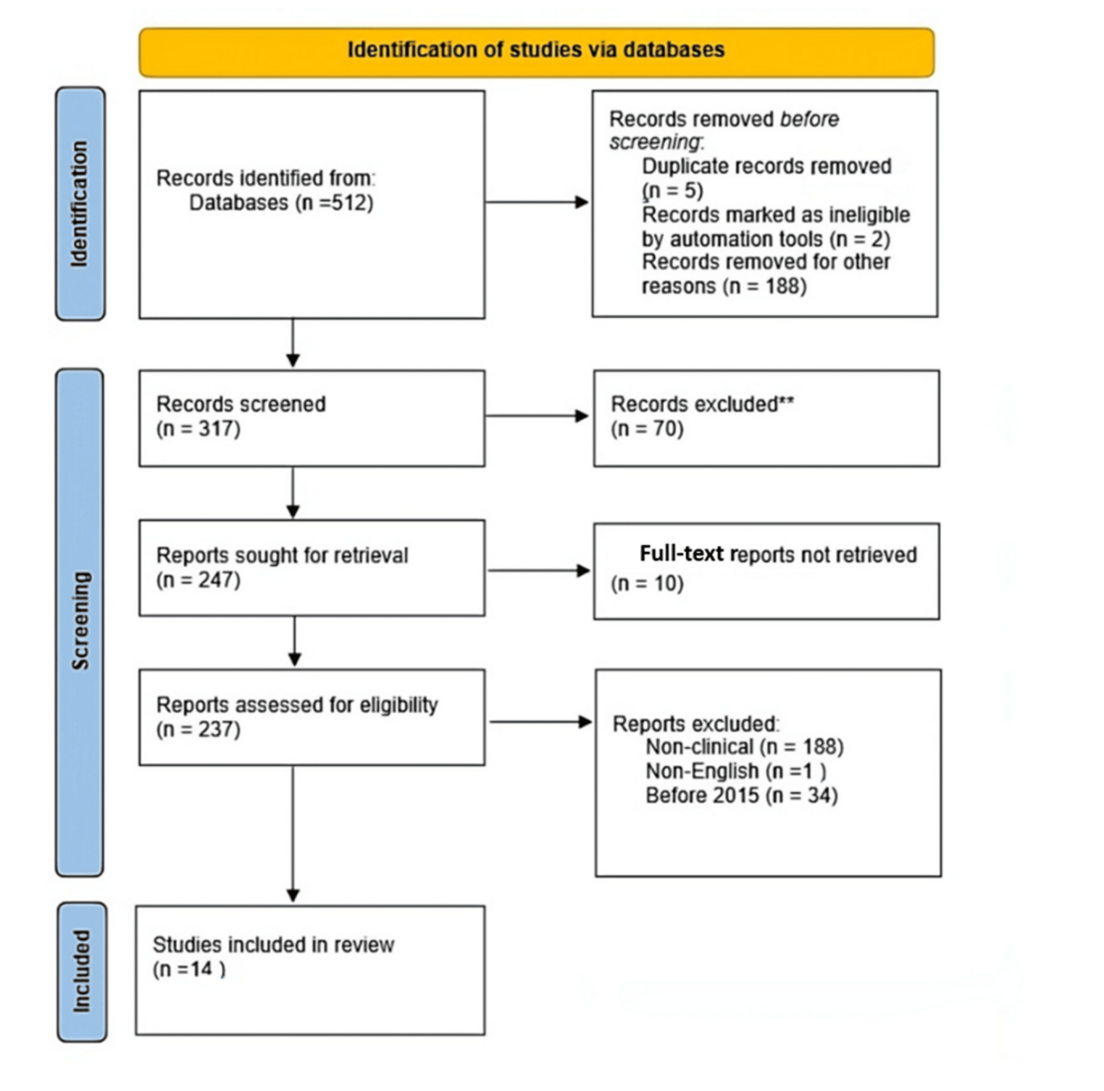
Preferred Reporting Items for Systematic Reviews and Meta-Analyses (PRISMA) 2020 flow diagram of study selection.

Ultimately, 14 studies met the inclusion criteria and were included in the qualitative synthesis. The study selection process is illustrated in Figure 1 (Preferred Reporting Items for Systematic Reviews and Meta-Analyses (PRISMA) flow diagram).

Inclusion and Exclusion Criteria

Studies were eligible for inclusion if they were published in English between January 2015 and August 2025; focused on AI or machine-learning applications for the diagnosis of knee lesions; utilized medical imaging data-primarily MRI, but also CT, radiography, or ultrasound; reported quantitative diagnostic performance metrics (e.g., sensitivity, specificity, accuracy, or area under the ROC curve (AUC)); employed validated reference standards (arthroscopy, radiologist consensus, or established grading systems); and included human subjects or clinically relevant datasets.

Studies were excluded: if they lacked quantitative diagnostic performance metrics; if conference abstracts without full-text availability were involved; in case of review articles without original data; in studies focused solely on image reconstruction or enhancement without diagnostic applications; in studies that were non-clinical or purely technical without medical relevance; or provided insufficient methodological detail for quality assessment (Figure 1).

Quality Assessment

The methodological quality of included studies was evaluated using adapted criteria from the Quality Assessment of Diagnostic Accuracy Studies (QUADAS-2) framework and the recently published TRIPOD+AI reporting guidelines for prediction models using AI [10]. Assessment criteria included study design and patient selection methodology; the appropriateness and implementation of the reference standard; execution and interpretation of the index test; risk of bias; applicability concerns; statistical analysis rigor; and validation methodology, distinguishing between internal and external validation.

Operationalization of Quality Assessment (QUADAS-2 and TRIPOD+AI)

The QUADAS-2 framework was operationalized by adapting its four core domains-patient selection, index test, reference standard, and flow and timing-to the context of AI-based diagnostic studies. Each domain was assessed for risk of bias as “low,” “high,” or “unclear” based on predefined criteria.

For patient selection, studies were considered low risk if they included representative patient populations with clear inclusion and exclusion criteria, while studies using highly selective or convenience datasets (e.g., single-center or publicly curated datasets without external validation) were rated as higher risk.

For the index test (AI model), low risk was assigned to studies that clearly described model architecture, training procedures, validation strategies, and avoided data leakage. Studies lacking transparency in model development or using inadequate validation methods (e.g., absence of external validation) were considered at higher risk.

For the reference standard, low risk required the use of clinically accepted gold standards, such as arthroscopy, expert radiologist consensus, or validated grading systems. Studies relying on less robust or poorly described reference standards were rated as having a higher risk.

For flow and timing, studies were rated as low risk if all patients received the same reference standard and if there was no inappropriate exclusion of cases. Inconsistent application of reference standards or incomplete reporting led to higher or unclear risk ratings.

Applicability concerns were assessed across three domains (patient selection, index test, and reference standard) based on how well each study reflected real-world clinical settings. Studies using highly controlled datasets, non-representative populations, or experimental conditions without clinical validation were considered to have higher applicability concerns.

In addition, elements from the TRIPOD+AI guidelines were incorporated to evaluate reporting transparency, particularly regarding model development, validation, and performance reporting.

Summary of Risk-of-Bias Trends

Overall, the included studies demonstrated moderate methodological quality. The most common sources of bias were related to patient selection (predominantly single-center or retrospective datasets) and index test evaluation (limited use of external validation and potential risk of overfitting).

The reference standard domain generally showed lower risk of bias, as most studies employed established clinical or imaging-based standards. However, variability in annotation quality and lack of standardized labeling protocols were noted in some studies.

With respect to applicability, several studies exhibited moderate to high concerns, primarily due to limited dataset diversity and the use of controlled experimental conditions that may not fully reflect routine clinical practice.

Data Extraction

Standardized data extraction forms were used to capture key study characteristics, including the study design and setting; patient demographics and sample size; imaging modality and acquisition parameters; AI/ML methodology and model architecture; the reference standard used; reported performance metrics (e.g., sensitivity, specificity, accuracy, AUC, F1-score); comparisons with human performance, where available; and considerations related to clinical implementation.

Scope and Focus

This review specifically focused on AI/ML applications for diagnosing knee lesions from approximately 2015 onwards to capture contemporary developments in deep learning and modern AI architectures. The temporal focus ensures relevance to current clinical practice while encompassing the period of significant advancement in medical AI applications. Studies were categorized by target pathology (ACL injuries, meniscal tears, cartilage lesions, osteoarthritis), AI methodology employed, and diagnostic performance metrics to facilitate comprehensive analysis and comparison.

Discussion

AI/ML Approaches for Knee Lesion Diagnosis

The landscape of AI applications in knee lesion diagnosis has been dominated by deep learning architectures, particularly convolutional neural networks (CNNs), which have demonstrated superior performance in medical image analysis tasks. A systematic analysis of 14 studies revealed that ResNet architectures were employed in 21% of investigations, followed by VGG networks (11%), DenseNet (8%), and various custom architectures [6]. These deep learning models have shown remarkable capabilities in automatically extracting relevant features from medical images without requiring manual feature engineering. (Table 1). Details of these studies are presented in Table 2.

**Table 1:** Architectures used across the reviewed corpus and representative use cases. Risk-of-bias assessments (e.g., low, moderate, high) were determined using adapted QUADAS-2 criteria, as described in the Methods section, considering patient selection, model development and validation, reference standards, and study design. ACL: anterior cruciate ligament, OA: osteoarthritis, MRI: magnetic resonance imaging, ResNet: residual network, VGG: Visual Geometry Group, MRNet: magnetic resonance network, DenseNet: densely connected convolutional network, U-Net: U-shaped convolutional neural network, Mask R-CNN: mask region-based convolutional neural network, 3D CNNs: three-dimensional convolutional neural networks

Architecture / family	Usage in corpus (%)	Representative use in knee imaging
ResNet	21	Classification/diagnosis across ACL, meniscus, OA
VGG	11	Baseline CNNs for MRI lesion classification
DenseNet	8	Feature-reuse CNNs for segmentation/classification
Other / Custom (incl. MRNet, U-Net, Transformers, Mask R-CNN, multi-task & 3D CNNs)	60	Multi-task learning, detection/segmentation, federated and transformer approaches

**Table 2:** Baseline characteristics of the included studies AUC-ROC: receiver operating characteristic-area under curve, OA: osteoarthritis, MRI: magnetic resonance imaging, CNN: convolutional neural network, SDMT: Spatial Dependence Multi-task Transformer, ACL: anterior cruciate ligament, PFJ: patellofemoral joint, ML: machine learning, DL: deep learning, PET/MR: positron emission tomography/magnetic resonance, MRAC: MR attenuation correction

Study and year	Design	Sample size	Main findings
Mead et al., 2024 [6]	Systematic review (retrospective, diagnostic accuracy / comparative analyses)	54 individual studies included	Deep learning (DL) models for knee MRI showed promising performance (sensitivity ~88.65%, specificity ~90.12%, AUC-ROC ~92.05%, accuracy ~88.30%). Specialised pathology-specific models outperformed general abnormality detection by up to ~4.5%
Yeoh et al., 2023 [11]	Development and evaluation of a 3D efficient multi-task neural network for knee OA diagnosis using MRI	Data from Osteoarthritis Initiative (exact N not stated)	Proposed a 3D multi-task CNN improving diagnostic accuracy for OA grading and lesion detection while reducing computation time.
Li et al., 2023 [12]	Development of the SDMT network for 3D knee MRI segmentation and landmark localization	Not specified	Introduced the Spatial Dependence Multi-Task Transformer, achieving high accuracy in both segmentation and anatomical landmark localization.
Namiri et al., 2020 [13]	Deep learning model for ACL injury severity staging from MRI	ACL-injured knees from the multi-institution dataset (N not stated)	Achieved accurate hierarchical grading of ACL injuries, outperforming the radiologist baseline in some categories.
Kumar et al., 2021 [14]	Three-stream multi-channel CNN with Laplacian filter for knee MRI abnormality detection	Not stated	Laplacian filter integration improved abnormality detection performance in MRIs compared to the baseline CNN.
Goel et al., 2025 [15]	Federated learning for knee injury diagnosis using few-shot learning	Multi-center data (N not stated)	Demonstrated federated few-shot learning enables accurate diagnosis across sites without sharing patient data.
Bien et al., 2018 [16]	Development and retrospective validation of MRNet for knee MRI interpretation	1,370 exams for training, 120 for validation	Deep-learning-assisted model improved detection of ACL tears, meniscal tears, and abnormal findings.
Astuto et al., 2021 [17]	Deep learning for automatic detection and grading of knee MRI abnormalities	4,900 exams from multiple centers	Achieved high accuracy for detecting and grading multiple knee abnormalities.
Couteaux et al., 2019 [18]	Mask-RCNN for automatic meniscus tear detection and classification	1,103 MRI exams	The model achieved high accuracy for tear detection and orientation classification.
Pedoia et al., 2019 [19]	3D CNN for detection and staging of meniscus and PFJ cartilage degeneration	576 subjects	High performance in staging degenerative changes in OA and ACL-injured knees.
Raza et al., 2024 [20]	Comparative study of ML classifiers for knee OA diagnosis	OAI dataset (N not stated)	Random forest and gradient boosting showed superior OA classification accuracy compared to other classifiers.
Listone et al., 2025 [21]	3D CNN for bone segmentation in low-field knee MRI	Not specified	Achieved accurate bone segmentation despite low-field MRI limitations.
Herpe et al., 2026 [22]	Impact of AI assistance on radiologists' knee MRI interpretation	200 MRI cases	AI assistance significantly improved radiologist sensitivity and reduced reading time.
Liu et al., 2018 [23]	DL-based MR attenuation correction for PET/MR	64 patients	Improved attenuation correction quality in PET/MR imaging compared to conventional MRAC methods.

Convolutional Neural Networks and Architecture Evolution

The evolution of CNN architectures for knee imaging has progressed from relatively simple networks to sophisticated multi-task and attention-based models. Three-dimensional CNNs have gained particular advantage for volumetric MRI analysis, enabling comprehensive assessment of entire joint structures. Recent investigations have demonstrated that 3D CNN approaches, such as the OA_MTL (Osteoarthritis Multi-Task Learning) model, can simultaneously perform knee structure segmentation and osteoarthritis classification with an accuracy of 82.5% for classification and a Dice similarity coefficient of 91.5% for segmentation tasks [11].

Advanced architectures using attention mechanisms and transformer-based approaches have emerged as promising alternatives to traditional CNNs. The Spatial Dependence Multi-task Transformer (SDMT) network, for instance, achieved 83.91% accuracy in segmentation tasks and 2.12 mm mean radial error in landmark localization, demonstrating superior performance compared to conventional single-task methods [12].

Multi-modal and Multi-task Learning

Contemporary AI approaches increasingly embrace multi-modal data integration, combining information from different MRI sequences, imaging planes, and even multiple imaging modalities. Studies have shown that sagittal plane imaging provides optimal diagnostic accuracy for ACL pathology, while coronal views offer advantages for meniscal assessment [13]. Multi-channel CNNs that simultaneously process axial, sagittal, and coronal views have achieved accuracies exceeding 92% in knee injury detection, significantly outperforming single-plane approaches [14].

The integration of federated learning approaches has emerged as a solution to data privacy concerns while enabling collaborative model development across multiple institutions. Recent research demonstrated that federated few-shot learning frameworks could achieve accuracies of 85.3% (axial), 82.1% (sagittal), and 71% (coronal) for multi-label knee injury classification, while maintaining patient data privacy through distributed learning protocols [15].

The performance metrics and diagnostic accuracy are presented in Table 3.

**Table 3:** Diagnostic performance by pathology/task AI: artificial intelligence, ACL: anterior cruciate ligament, AUC: area under the curve, KL: Kellgren-Lawrence, OA MRI: osteoarthritis magnetic resonance imaging

Pathology/task	Reported AI performance (range or representative)	Notes
ACL tear detection (classification)	Accuracy 86.7–100%; AUC up to 0.965; sensitivity 89–93%; specificity 88–90%	AI is strong even for subtle/partial tears in some studies
Meniscal tear detection (localization/classification)	Accuracy 72.5–90%; AUC ≈ 0.90; sensitivity ≈89.8%; specificity ≈82.0% (dataset-dependent)	Performance improves with high-quality consensus annotations
Osteoarthritis (KL grading from radiographs)	Accuracy 95.7–98.9% (e.g., XGBoost models)	High performance across severity grades reported
Cartilage/OA MRI (segmentation and staging)	Segmentation Dice ≈0.915 (knee structures); bone segmentation Dice ≈0.984; classification accuracy ≈82.5%	3D CNNs and transformers are emerging; metrics vary by task and dataset.

ACL Injury Detection

AI systems have demonstrated exceptional performance in ACL injury detection, with accuracy rates ranging from 86.7% to 100% across multiple studies. The MRNet architecture, specifically designed for knee MRI analysis, achieved an area under the curve (AUC) of 0.965 for ACL tear classification on internal datasets [16]. More recent investigations utilizing advanced 3D CNN architectures have reported sensitivities of 89% to 93% and specificities of 88% to 90% for ACL tear detection, comparable to experienced musculoskeletal radiologists [17].

Notably, AI systems have shown particular strength in detecting subtle or partial ACL tears that may be challenging for human interpretation. Custom CNN architectures incorporating Laplacian filtering have achieved 92.8% accuracy in distinguishing between intact ACL, partial tears, and complete ruptures, demonstrating superior performance compared to traditional diagnostic approaches [14].

Meniscal Pathology Assessment

Meniscal tear detection represents a more challenging diagnostic task due to the complex anatomy and varied tear patterns. AI performance for meniscal pathology has shown variability across studies, with accuracies ranging from 72.5% to 90%. The Mask R-CNN framework has demonstrated particular efficacy in meniscal lesion localization and characterization, achieving AUCs of 0.90 for tear detection [18].

Recent studies have highlighted the importance of training data quality and annotation consistency for meniscal pathology detection. Models trained on high-quality, consensus-annotated datasets have achieved significantly better performance, with sensitivities of 89.81% and specificities of 81.98% for meniscal tear detection [19].

Osteoarthritis Grading and Cartilage Assessment

AI applications in osteoarthritis assessment have shown remarkable success, particularly in automating Kellgren-Lawrence (KL) grading from radiographs and assessing cartilage deterioration from MRI. Machine learning models utilizing XGBoost algorithms have achieved 98.90% accuracy in distinguishing between healthy and osteoarthritic knees, with excellent performance across all severity grades [20].

**Figure 2:**
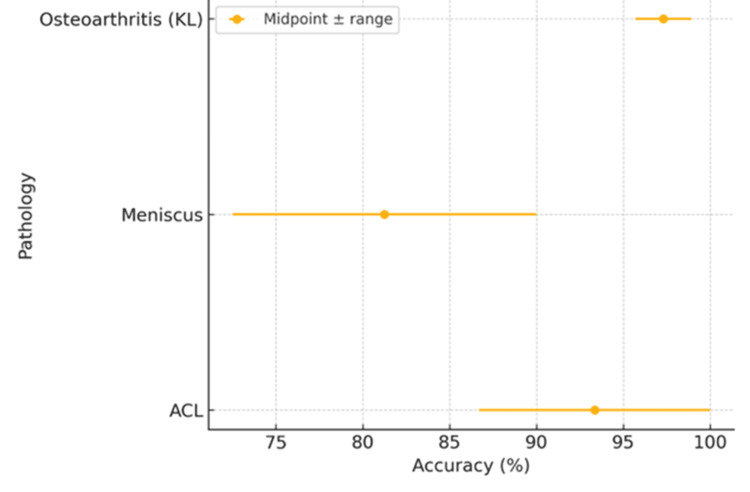
Reported accuracy ranges by pathology from the selected studies Anterior cruciate ligament (ACL) 86.7–100%, meniscus 72.5–90%, and osteoarthritis Kellgren-Lawrence (KL) grading 95.7–98.9%. Points denote midpoints; error bars span the reported min–max.

Advanced deep learning approaches for cartilage assessment have demonstrated capabilities in detecting subtle changes that may precede radiographically evident osteoarthritis. Three-dimensional U-Net architectures applied to low-field MRI have achieved Dice similarity coefficients of 0.9838 for bone segmentation, enabling precise quantification of joint space narrowing and osteophyte formation [21] (Figure 2).

Comparison with Radiologist Performance

Diagnostic accuracy comparison: Direct comparisons between AI systems and radiologist performance have yielded encouraging results, particularly in the context of AI-assisted interpretation rather than autonomous diagnosis. A recent multi-reader, multi-case study involving six fellowship-trained musculoskeletal radiologists with varying years of experience demonstrated that AI assistance improved diagnostic sensitivity from 81% to 86% and overall accuracy from 86% to 91% for knee lesion detection [22]. Specificity also improved from 88% to 93% with AI support, while inter-reader agreement increased substantially (Fleiss' Kappa from 54% to 78%). These findings suggest that AI can enhance both individual and collective diagnostic performance when used as a decision-support tool.

**Figure 3:**
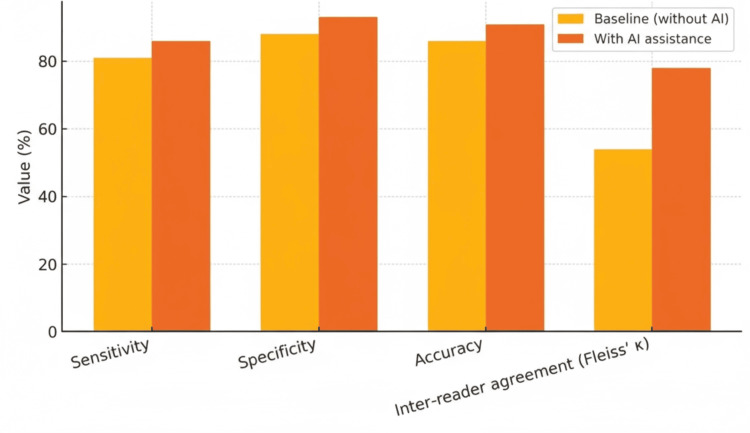
Effect of AI assistance on reader performance in a multi-reader study Sensitivity 81%→86%, specificity 88%→93%, accuracy 86%→91%, and inter-reader agreement (Fleiss’ κ) 54%→78%

Studies comparing standalone AI performance with that of fellowship-trained musculoskeletal radiologists have reported comparable-and in some cases higher-diagnostic metrics for specific tasks such as ACL tear detection. Reported AI sensitivities range from 92% to 94%, compared to 85% to 90% for radiologists, with similar or slightly improved specificity [23]. However, these comparisons are often derived from retrospective datasets under controlled conditions, and variability in reader expertise, case complexity, and study design should be considered when interpreting these results. Importantly, current evidence predominantly supports the role of AI in augmenting radiologist performance rather than replacing clinical judgment (Figure 3).

Workflow integration and efficiency: Beyond diagnostic accuracy, AI systems offer potential advantages in workflow efficiency and consistency, particularly when integrated into clinical decision-making pathways. AI-assisted interpretation has been reported to reduce reading time for knee MRI studies from an average of 15-25 minutes to approximately five to eight minutes, depending on case complexity and reader experience [24]. These gains are most relevant in high-volume clinical settings and may help alleviate increasing workload demands on radiologists.

Another important advantage is the potential for improved consistency. While human interpretation may be influenced by fatigue, experience level, and case load, AI systems provide standardized outputs across repeated evaluations. Studies have demonstrated lower intra-observer variability in AI-assisted settings compared to unaided human interpretation [25]. Nevertheless, optimal performance is achieved when AI outputs are interpreted within the clinical context by experienced radiologists, reinforcing the concept of AI as a complementary tool.

Limitations and Challenges

Dataset diversity and generalizability: One of the most significant limitations in current AI applications is the lack of dataset diversity. Most training datasets are derived from single institutions or limited demographic populations, potentially limiting model generalizability across different patient populations, imaging protocols, and clinical settings. Studies have shown that AI models may perform suboptimally when applied to datasets with different demographic characteristics or imaging parameters than those used during training [26].

The predominance of studies utilizing 2D MRI data rather than 3D volumetric approaches represents another limitation. While 2D models have achieved higher average specificity and accuracy metrics, the integration of 3D volumetric information offers theoretical advantages for comprehensive joint assessment [25].

Model interpretability and clinical trust: The limited interpretability of deep learning models presents a major barrier to their integration into clinical practice. Healthcare professionals require an understanding of diagnostic decision-making processes to maintain clinical oversight and patient safety. Recent developments in explainable AI, including gradient-weighted class activation mapping (Grad-CAM) and attention visualization techniques, offer promising approaches to enhance model interpretability [27].

Studies have identified that a lack of trust in AI recommendations represents a major barrier to clinical adoption. Healthcare professionals express concerns about algorithmic bias, model reliability in edge cases, and the potential for automation bias affecting clinical decision-making [28].

Regulatory and ethical considerations: The regulatory landscape for AI-enabled medical devices continues to evolve, with the FDA implementing new frameworks for AI/ML software as medical devices. Current regulatory requirements emphasize the need for robust validation studies, bias mitigation strategies, and post-market surveillance protocols [29]. The recent FDA guidance on predetermined change control plans for AI/ML-enabled devices provides a framework for managing model updates and improvements while maintaining regulatory compliance. Ethical considerations include ensuring algorithmic fairness across diverse patient populations, maintaining patient privacy during model development, and addressing potential conflicts of interest in AI development and deployment [30].

Clinical integration challenges: The integration of AI tools into existing clinical workflows presents multiple technical and logistical challenges. Interoperability with existing picture archiving and communication systems (PACS), electronic health records, and imaging equipment requires significant technical infrastructure investment [31]. Additionally, the need for ongoing model validation, performance monitoring, and maintenance presents operational challenges for healthcare institutions. Training requirements for healthcare professionals represent another implementation barrier. Successful AI integration requires comprehensive education programs to ensure appropriate utilization and interpretation of AI recommendations within clinical contexts [32].

Future Directions and Research Gaps

Multimodal integration and advanced architectures: Future developments in AI for knee lesion diagnosis will likely emphasize multimodal data integration, combining imaging data with clinical history, physical examination findings, and laboratory biomarkers. Graph neural networks and transformer architectures show particular promise for integrating diverse data types and capturing complex relationships within joint pathology [33]. The development of foundation models and large language models specifically trained on medical imaging data may enable more sophisticated diagnostic capabilities and improved generalizability across different clinical scenarios [34].

Real-world validation and deployment: There remains a significant gap between controlled research environments and real-world clinical deployment. Future research must prioritize large-scale, multicenter validation studies that encompass diverse patient populations, varied imaging protocols, and different clinical settings. Prospective studies evaluating AI performance in routine clinical practice are essential for demonstrating real-world effectiveness and safety [35].

Personalized medicine and risk prediction: Beyond diagnostic applications, AI technologies offer potential for personalized risk prediction and treatment planning. Machine learning models capable of predicting disease progression, treatment response, and long-term outcomes could significantly enhance clinical decision-making and patient counseling [36]. The integration of AI with emerging imaging techniques, including quantitative MRI, advanced diffusion imaging, and novel contrast mechanisms, may enable earlier detection of pathological changes and more precise disease characterization [37].

## Conclusions

AI and ML have made great strides in knee lesion diagnosis over the past decade, with current systems approaching or exceeding human accuracy across multiple pathological conditions. The most popular method is DL architectures, particularly CNNs, which can detect ACL injuries (86.7-100% accuracy), grade osteoarthritis (95.7-98.9% accuracy), and assess meniscal pathology. When compared to experienced radiologists, AI-assisted diagnosis improves sensitivity (81% to 86%), accuracy (86% to 91%), inter-reader agreement, interpretation time, and specificity. Before widespread clinical adoption, several critical issues must be addressed. These include dataset diversity and model generalizability issues, model interpretability and transparency issues, regulatory requirements, and clinical workflow integration barriers. The prevalence of single-center studies and limited demographic diversity in training datasets raises concerns about equitable performance across diverse patient populations.

Looking ahead, the field must focus on large-scale, multicenter validation studies that demonstrate real-world clinical effectiveness, diverse, representative training datasets that ensure equitable performance across patient populations, explainable AI techniques that increase clinical trust and adoption, standardized model development protocols, and valid

It appears that AI and ML technologies will become more important in knee lesion diagnosis, improving diagnostic accuracy, workflow efficiency, and patient outcomes. Clinicians, researchers, technology developers, and regulatory authorities must work together to overcome current limitations and ensure safe, effective, and equitable use of these powerful diagnostic tools.
